# Impact of the COVID-19 Pandemic on Lung Function and Treatment Decisions in Children with Asthma: A Retrospective Study

**DOI:** 10.3390/jcm14103289

**Published:** 2025-05-08

**Authors:** Jaqueline Abdul-Razzak, Mihaela Ionescu, Radu Diaconu, Alexandru Dan Popescu, Elena Carmen Niculescu, Ileana Octavia Petrescu, Cristina Elena Singer, Carmen Simona Coșoveanu, Liliana Anghelina, Cristian Gheonea

**Affiliations:** 1 Doctoral School, University of Medicine and Pharmacy of Craiova, 200349 Craiova, Romania; jaquelineabdulrazzak90@gmail.com; 2 Department of Pediatrics “Mother and Child”, Faculty of Medicine, University of Medicine and Pharmacy of Craiova, 200349 Craiova, Romania; carmen.niculescu@umfcv.ro (E.C.N.); ileana.petrescu@umfcv.ro (I.O.P.); cristina.singer@umfcv.ro (C.E.S.); simona.cosoveanu@umfcv.ro (C.S.C.); liliana.anghelina@umfcv.ro (L.A.); cristian.gheonea@umfcv.ro (C.G.); 3 Department of Medical Informatics, Faculty of Dental Medicine, University of Medicine and Pharmacy of Craiova, 200349 Craiova, Romania; 4 Department of Endodontics, Faculty of Dental Medicine, University of Medicine and Pharmacy of Craiova, 200349 Craiova, Romania; alexandrudanpopescu20@gmail.com

**Keywords:** pediatric asthma, FeNO, pulmonary function test, SARS-CoV-2, treatment adherence, parental management

## Abstract

**Background/Objectives**: Asthma outcomes in children and adolescents largely depend on parental adherence to prescribed treatment plans. This study investigates how the COVID-19 pandemic influenced parental decision-making in managing their children’s asthma, regardless of whether the children were infected with SARS-CoV-2. **Material and method**: In this research, 146 children with asthma were analyzed based on the following data: demographic parameters (gender, age group, and residence), before and after measurements of FeNO and pulmonary function test parameters were performed to assess the evolution of asthma for infected and non-infected children, exacerbations, parents’ compliance with the treatment, changes in treatment steps performed by physicians, and the GINA asthma control levels. **Results**: The effect of parent self-management of doses was evident in the variation of FeNO and pulmonary function test parameters before and after COVID-19 disease, including children with asthma who did not contract the virus, in the decrease in well-controlled asthma cases, as well as in the number of exacerbations per year. A step-down in treatment doses was statistically associated with increased FeNO values (*p* < 0.0005), and decreased FEV1 values (*p* = 0.025). Higher values of FeNO were statistically significantly associated with a higher number of exacerbations per year (*p* < 0.0005). There was a statistically significant moderately strong association between the treatment steps evolution (decided by the attending physician) and parents’ self-management of doses in the attempt to assess the control of the disease of children with asthma (*p* = 0.019). Also, 80.95% of children for whom the parents performed a step-down in dose no longer presented well-controlled asthma, leading to a statistically significant association relative to the level of asthma control and doses adjustments (*p* < 0.0005). **Conclusions**: During epidemiological circumstances, a strong collaboration between the parents/caregivers/pediatric patients with asthma and attending physicians is essential to correctly assess the symptoms and to comply the asthma treatment with ICS and a bronchodilator in order to control the disease.

## 1. Introduction

Asthma is one of the most common chronic respiratory pathologies in the pediatric population. This chronic lung disease can be managed by assessing lung function and controlling clinical symptoms, in particular wheezing, coughing, chest tightness and dyspnea. As of today, there are still difficulties in maintaining the well-being of these patients, with frequent exacerbations being the reason for presentation to emergency departments. Certainly, asthma can be managed by medical follow-ups, adherence to the prescribed medication according to the type of asthma that pediatric patients present with, as well as avoiding exposure to triggers [1,2,3,4,5].

The less controlled the asthma, the higher the frequency of exacerbations, and this has been demonstrated by the presence of predisposing and favoring factors such as young age, unfavorable socioeconomic status, exposure to viral agents or pollutants, and noncompliance with the doses of medication recommended by the treating physician in the treatment of asthma [3,6].

Although a cause of concern for health care professionals, the COVID-19 pandemic period kept pediatric patients with asthma away from viral or allergic triggers during the early part of the pandemic. However, some patients felt that ICS (inhaled corticosteroid) medical devices were a means of spreading infection and lowering immunity and they discontinued their basic treatment and stopped following their physician’s recommendations [3,6,7,8,9,10].

Optimal adherence can be achieved by combining therapy for asthma exacerbation episodes (short-term relief medications) and preventive therapy (long-term relief medications), thus achieving better asthma control [11,12,13]. Pediatric patients who receive only SABA (short-acting beta-agonists bronchodilator) as needed are at risk of increased bronchial hyperresponsiveness and thus poorer asthma control, with the number of SABA doses used being a factor in monitoring adherence to treatment as well as the number of exacerbations [8,14,15,16,17]. According to the GINA strategy report guideline, it is recommended to administer ICS–formoterol combination in low dose as a combination of maintaining and reliever therapy (MART) for asthma to lower the occurrence of exacerbations and the number of emergency department presentations compared to using SABA only as needed or ICS daily [18].

The COVID-19 pandemic period impacted the pediatric population, especially children with chronic conditions such as asthma [19,20,21]. Initially, at the onset of the pandemic, it was thought that corticosteroids lowered the immune system of patients so that they would have been prone to increased risk of SARS-CoV-2 infection, which made parents or caregivers decrease or completely remove the controller medication of children with asthma in order not to be at risk. However, no correlation was found between ICS and contracting SARS-CoV-2 infection, with the GINA guidance about COVID-19 stating that throughout the pandemic children with asthma should adhere to corticosteroid therapy, with the decision to step-down or discontinue the treatment not being an appropriate one [22,23].

Another important aspect is that adolescents and children are dependent on parental decisions. The lockdown during COVID-19 pandemic affected pediatric patients with asthma emotionally because they had to comply with much stricter conditions imposed by their parents, creating a higher vulnerability to stress [24,25]. The welfare of these patients depends very much on adherence to the control treatment prescribed by the attending physician, on the parents’ ability and decisions to manage the disease, on their understanding of the children’s needs, and on their compliance with the prescribed dose without being influenced by certain epidemiologic circumstances, i.e., lowering or abuse of the control treatment dose without sound medical reasons. Compliance with these recommendations will not only improve the relationship between children or adolescents and their parents, relatives, and friends, but will also generate a lower risk of developing exacerbations, giving them the confidence and security of having a comfort level correlated with the pathology they are affected by [25,26,27,28,29,30].

This study provides topical information on the impact that the decisions of parents of children with asthma have had on their maintenance and reliever treatment and on how the COVID-19 pandemic period has influenced disease management. Although it is a chronic lung disease that can be kept under control, there are still some discrepancies that could compromise the well-being of pediatric patients with asthma. The effects that infection with SARS-CoV-2 virus have had on children with diagnosed asthma are still debated and little data in the field of expertise emphasize the impact that the COVID-19 pandemic has had on them. It may be regarded as certain that communication with the physician, attention to the needs of pediatric patients with asthma, and ensuring regular medical follow-ups should not be changed regardless of what special epidemiological conditions will occur in the future.

The aim of this study is to evaluate how the COVID-19 pandemic influenced parental decisions in pediatric asthma management using clinical and paraclinical data, addressing a relevant timeline issue and a current topic.

## 2. Materials and Methods

### 2.1. Study Design and Participants’ Selection

A retrospective study was performed in the Pediatric Department of a Regional Tertiary Hospital (Filantropia Clinical Municipal Hospital Craiova, Romania), between March 2020–July 2024 on children with diagnosed asthma who benefited from periodic follow-ups. Pulmonary function tests values and the fraction of exhaled nitric oxide (FeNO) values were used both before and after infection with SARS-CoV-2 in relation to asthma treatment to highlight changes in lung function in pediatric patients previously diagnosed with asthma and for those who did not contract the virus. Additionally, before and after measurements were used to assess the evolution of asthma over time.

Values of FeNO, pulmonary function test parameters, and the level of asthma control at regular follow-ups visits were correlated with treatment modification of children with asthma. Control of asthma was assessed based on GINA guidelines and variation in treatment steps before and after SARS-CoV-2 infection compared to children with asthma who did not contract the virus.

FeNO and pulmonary function test parameters were measured from all pediatric patients with asthma involved in the study, regardless of whether the children were infected with SARS-CoV-2.

FeNO was measured using Aerocrine Niox Vero 12-1000 analyzer (NIOX Group plc, Uppsala, Sweden) and the results were reported in ppb (parts per billion).

Spirometry was performed using the Vitalograph Pneumotrac 6800 spirometer (Vitalograph, Hamburg, Germany) which determined the following pulmonary function parameters: FVC (forced vital capacity), FEV1 (forced expiratory volume in the first second), PEF (peak expiratory flow), and FEF_25–75_ (forced mid expiratory flow).

A positive SARS-CoV-2 infection was identified through a PCR test or through rapid antigen test.

To conceptualize this study, inclusion criteria were structured as stated below:

- Pediatric patients with asthma who are less than 18 years old whose parents/legal guardians agreed to participate in the study;
- Children with asthma who received treatment steps according to the GINA guideline;
- Children with asthma who were assessed according to the GINA guideline to identify the level of asthma control;
- Pediatric patients with asthma who had pulmonary function test values and FeNO values measured before and after infection with the SARS-CoV-2 virus;
- Pediatric patients with asthma who did not contract the virus and had before and after measurements of pulmonary function test values and FeNO values to assess the evolution of asthma over time.
- Exclusion criteria were as follows:
- Children with asthma who present other chronic pathologies that may intervene with the results of the present study;
- Pediatric patients with asthma who, prior to infection with SARS-CoV-2, did not measure pulmonary function test parameters or FeNO;
- Pediatric patients with asthma who did not contract the virus and did not have before and after measurements of pulmonary function test values and FeNO values to assess the evolution of asthma over time.

The study was approved by the Ethics Committee of the University of Medicine and Pharmacy of Craiova, no. 167/14.09.2023 and it respected the Declaration of Helsinki. All subjects’ parents or legal guardians signed an informed consent form on behalf of pediatric patients.

### 2.2. Study Variables

**Figure 1:**
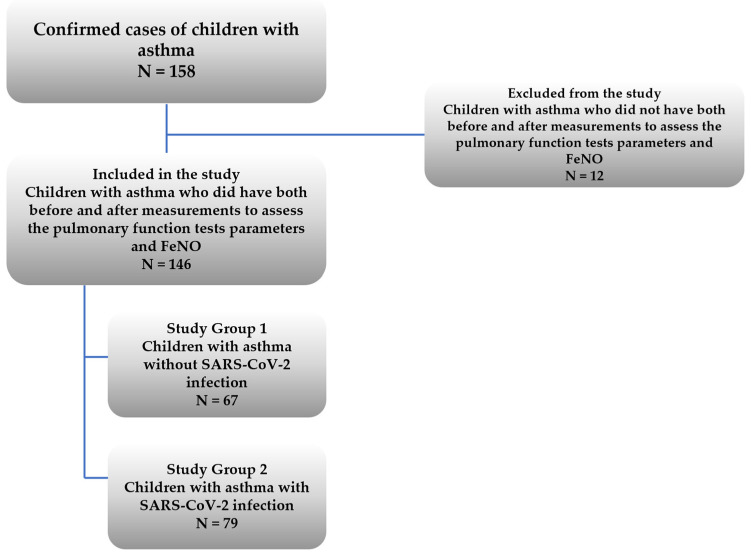
The structure of the study group.

COVID-19 and non-COVID-19 evaluates the two pediatric patients with asthma included in the study group meaning patients who have contracted the infection (Study Group 2: with SARS-CoV-2 infection, N = 79) and those who did not contract the virus (Study Group 1: without infection, N = 67) (Figure 1).

“Before” and “after” measurements study the entire study group (146 pediatric patients with asthma) evaluated in terms of pulmonary function test parameters, FeNO, exacerbations, and treatment steps.

FeNO values expressed as a continuous data series. Pulmonary function test parameters (FVC, FEV1, PEF, and FEF_25–75_) expressed as continuous data series.

Treatment step refers to the ordinal data from Step 1 to Step 5 according to the GINA guideline.

All children with asthma received standardized care and follow-up.

### 2.3. Statistical Analysis

Statistical tests were applied with SPSS (Statistical Package for Social Sciences) software, version 26 (SPSS Inc., Armonk, NY, USA). Continuous data series were evaluated for normality using the Kolmogorov–Smirnov/Shapiro–Wilk test. Following these results, which emphasized only non-normally distributed data, continuous series were described as median values. Nominal and ordinal variables were stated as frequency distributions and percentages. Comparisons between various groups on continuous variables were conducted using the Mann–Whitney U and Kruskal–Wallis H non-parametrical tests. Pairwise comparisons were performed using Dunn’s procedure, along with a Bonferroni correction performed for multiple comparisons. Associations were tested using the Chi-square test. *p*-values which are smaller than 0.05 represented statistically significant results.

## 3. Results

A total of 146 (90 boys) eligible pediatric patients with asthma were included in this study group (Study Group 1 and Study Group 2), meeting the inclusion and exclusion criteria (Figure 1). With a sample size of 146 participants, an effect size value of 0.25, and a β/α ratio of 1, the software application G*Power 3.1.9.7 (Heinrich Heine University Düsseldorf, Germany) computed the power of the study to be 0.90, which is above 0.80 which is the value typically accepted.

The study included children with asthma who are less than 18 years old, divided by gender as follows: 56 girls (38.36%) and 90 boys (61.64%). Relative to the residence area, the children included in the study were mostly from urban areas (104, 71.23%), and less than a quarter were from rural areas (42, 28.77%). According to age, the study group comprised children of various ages, for example, less than 6 years old (2 children, 1.37%), 6–11 years old (108 children, 73.97%), and 12–17 years old (36 children, 24.66%). The assessment according to the age at diagnosis led to the following age group divisions: children diagnosed at ages less than 6 years old (64 children, 43.84%), children diagnosed at ages between 6 and 11 years old (75 children, 51.37%), and children who had been diagnosed as teenagers at ages between 12 and 17 years old (7 children, 4.79%).

### 3.1. Baseline Lung Function and FeNO Measurements

The retrospective analysis on the FeNO values and pulmonary function test parameters measured before SARS-CoV-2 infection indicated that the study group was homogenous with respect to the demographic data. According to a Mann–Whitney U test, there were no differences in FeNO and pulmonary function test parameters by gender or residence. Distributions of these parameters for children within those categories were similar, as assessed by visual inspection. Median FeNO and median pulmonary function test parameters were not statistically significantly different between girls and boys, or between children from urban and rural areas, *p* > 0.05 (Table 1).

Analysis based on a Kruskal–Wallis test was conducted to determine if there were differences in FeNO and pulmonary function test parameters between different age groups. Distributions of FeNO and pulmonary function test parameters were similar for all three groups, as assessed by the visual inspection of a boxplot. Statistically significant differences between the different age groups were identified for the median FeNO values (χ^2^(2) = 14.372, *p* = 0.001) and median PEF variation values (χ^2^(2) = 8.042, *p* = 0.018) (Table 1).

Before the SARS-CoV-2 infection, children with asthma were under treatment at different medication steps (since there was only one child with Step 5, they were grouped with children in Step 4 for the ease of calculation). Analysis based on a Kruskal–Wallis test was conducted to identify the presence of differences in FeNO and pulmonary function test parameters between children with asthma with different treatment steps, but no statistically significant differences were identified, *p* > 0.05 (Table 2).

### 3.2. Follow-Up Parameters Measurements in Non-Infected (Study Group 1) and Infected (Study Group 2) Children with Asthma

During the first part of COVID-19 pandemic, safety measures included lockdown, restrictions on outdoor activities, and less exposure. Nevertheless, more than half of the children included in the study presented SARS-CoV-2 infection (79 children, representing 54.10% of the entire study group). Measurements of FeNO and pulmonary function test parameters reflected the same homogeneous study group regarding gender, residence, and age group (Table 3).

Statistically significant differences between the different age groups were identified for the median FeNO values (χ^2^(2) = 9.172, *p* = 0.010) and median PEF variation values (χ^2^(2) = 8.609, *p* = 0.014) (Table 3). The same variables presented statistically significant differences before COVID-19 disease too (Table 1). For FeNO, the smallest median value belonged to the group “less than 6 years old” (4.00), then increased as the age group increased: 23.50 for group “6–11 years old” and 31.00 for group “12–17 years old”. Subsequently, pairwise comparisons were conducted using Dunn’s procedure. A Bonferroni correction corresponding to multiple comparisons was made with a statistical significance threshold accepted at the value *p* < 0.0166 level. This post hoc type of analysis revealed statistically significant differences in FeNO values between the “less than 6” and “12–17 years old” (*p* = 0.007) groups, and the “6–11” and “12–17” years old (*p* = 0.002) groups, but not between the other group combination. For PEF variation, the smallest median belonged to the group “6–11 years old” (0.68), then increased for group “12–17 years old” (0.73) and then for group “less than 6 years old” (1.025). The post hoc analysis did not reveal any statistically significant differences in PEF variations values between groups (Table 3).

An analysis based on a Kruskal–Wallis test was conducted to identify the presence of differences in FeNO and pulmonary function test parameters between children with different doses adjustments (parents’ self-management of doses). Statistically significant differences were observed for median FeNO values as children with a step-down in dose had the highest median of 39.50, children with the same dose had a median of 20.50, while children with a step-up in dose had a median of 19.50 (χ^2^(2) = 36.682, *p* < 0.0005). A Bonferroni correction for multiple comparisons was made with statistical significance accepted at the value *p* < 0.0166 level. This post hoc analysis indicated statistically significant differences in FeNO values between the “step-down” and “same” groups (*p* < 0.0005), and between “step-down” and “step-up” groups (*p* < 0.0005), but not between the other group combination. FEV1 median values also decreased significantly between groups as children with a step-down in dose had a median value of 0.78, significantly lower than children with the same step with a median of 0.87, and children with a step-up in dose had a median of 0.85 (χ^2^(2) = 7.392, *p* = 0.025). The post hoc analysis emphasized statistically significant differences in FEV1 values between the “step-down” and “step-up” groups (*p* = 0.010), but not between the other group combinations (Table 4).

An overall analysis considering children with a treatment modification (so children with both a step-up and a step-down in their medication doses) provided similar results: children with a modification of treatment had a median FeNO value (29.50) significantly higher than children with no modification of treatment (19.50) *p* = 0.001 (Table 4).

For both study groups, the physician decided to assess the children with asthma and adjust their controller medication. So, there was a one step-down in dose for 10 children (representing 6.85%), for 135 children (92.47%) the same step of medication was maintained as before the parent/caregiver self-managed the medication doses for their children with asthma, and a one step-up in dose for 1 child (0.68%). Since there was only one child with a one step-up in dose, they were grouped with children with the same step for the ease of calculation (Table 5).

A Chi-square test was conducted between treatment step evolution and dose adjustment by the physician correlated to parents’ self-management of doses to assess the control of the disease of pediatric patients with asthma included in the study. There was a statistically significant moderately strong association between these parameters, χ^2^(1) = 7.913, *p* = 0.019, and φ = 0.233 (Table 5).

Parents’ decisions to adjust treatment doses impacted significantly the assessment of asthma control at clinical visits. Thus, 80.95% of children for whom the parents performed a step-down in dose ended up with partially controlled asthma, compared to only 19.05% who remained with well-controlled asthma. Maintaining the same step led to a majority of children being evaluated as having well-controlled asthma. Overall, the partially controlled asthma was associated with an adjustment of doses, mostly a step-down, χ^2^(2) = 24.800, *p* < 0.0005, and φ = 0.412 (Table 6).

The analysis regarding the distribution of FeNO and pulmonary function test parameters divided by the GINA asthma control levels revealed that children with partially controlled asthma have significantly higher values of FeNO compared to children with well-controlled asthma, U = 4349.50, z = 6.600, and *p* < 0.0005. There are no statistically significant differences for the other parameters between children with different GINA asthma control levels (Table 7).

Analysis based on the Mann–Whitney test was conducted to determine if there were differences in FeNO and pulmonary function test parameters among children with asthma whose parents decided to self-manage their children’s treatment or not, and how it influenced the physician’s decision to change pediatric patients with asthma treatment afterwards (Table 8). Statistically significant differences were observed for median FeNO values, as children with a one step-down in dose had the smallest median value of 9.00 and children with the same step as before their parents self-managed the asthma treatment of their children or had one step-up in dose had a median of 26.00 (U = 1021.50, z = 2.647, and *p* = 0.008). FEV1 median values also decreased significantly between groups as children with one step-down in dose had a median value of 0.915, significantly higher than children with the same step as before the parents self-managed the asthma treatment or had one step-up in treatment who had median of 0.820 (U = 308.50, z = −2.880, and *p* = 0.004).

#### Asthma Exacerbations and Their Association with Treatment Changes

Following the analysis of exacerbations for non-infected and infected children with asthma, it was noticed that 51 children (representing 34.93%) did not have any exacerbation, 56 children (38.36%) had one exacerbation per year, 31 children (21.23%) had two exacerbations per year, and 8 children (5.48%) had three exacerbations per year.

Similar analysis based on the Kruskal–Wallis test was conducted to identify the differences in FeNO and pulmonary function test parameters between children with various numbers of exacerbations per year for both study groups. Distributions of FeNO and pulmonary function test parameters were similar for all groups, as assessed by the visual inspection of a dedicated boxplot. Statistically significant differences between the different numbers of exacerbations were identified only for the median FeNO values (χ^2^(3) = 39.411, *p* < 0.0005), as children with no exacerbation per year had the smallest median FeNO value (11.00), followed by one per year (29.50), two per year, and three per year (each with 37.00) (Table 9).

The effect of parent self-management of doses was evident in the number of exacerbations. Almost half of children who remained with the same dose or received a step-up in dose had no exacerbation per year. A step-up in dose was associated with a reduction in the number of exacerbations per year, while a step-down in dose was associated with an increase in the number of exacerbations per year, as seen in Table 10. There was a statistically significant strong association between these parameters, χ^2^(6) = 36.383, Cramer’s V = 0.353, and *p* < 0.0005.

After the treatment step adjustment by the current physician, no statistically significant association was identified between the treatment and the number of exacerbations per year. Of the 10 patients with one step-down in dose, 6 (60.0%) had no exacerbation per year, and the other 4 had only one exacerbation per year. For patients with the same step or one step-up in dose, almost 40% had one exacerbation per year, and the percentage decreases for the other categories, according to Table 10.

### 3.3. Comparison Between Non-Infected and Infected Children with Asthma

**Figure 2:**
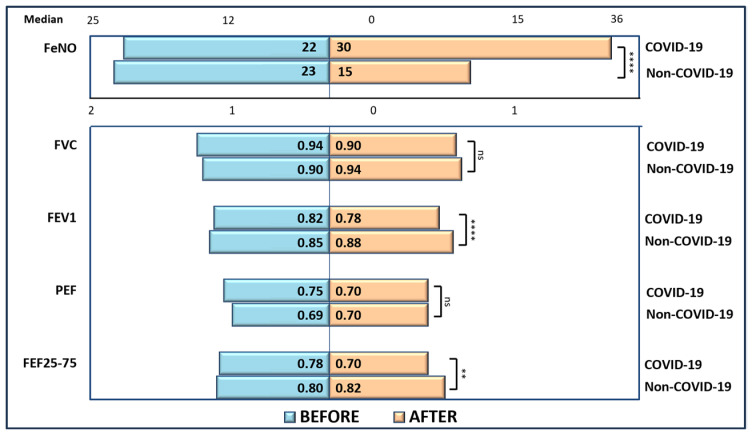
Baseline and follow-up evolution of FeNO and lung function parameters for Study Group 1 and Study Group 2. ns represents *p* > 0.05, ** represents *p* ≤ 0.01, **** represents *p* ≤ 0.0001.

A Mann–Whitney U test was run to determine the differences in FeNO and pulmonary function test parameters in both studied groups (i.e., children infected with SARS-CoV-2 virus and children who were not infected with the virus). Distributions of these parameters for children within those categories were similar, according to a visual inspection. Only PEF variation values were statistically significantly different between groups and the median PEF variation value for children who would later develop COVID-19 disease was initially significantly higher than the median PEF variation value for children not affected by COVID-19 disease, U = 1757.50, z = −3.493, and *p* < 0.0005 (Figure 2).

A similar test was conducted to determine if there were differences in FeNO and pulmonary function test parameters between children who were affected by SARS-CoV-2 infection or not. Distributions of these parameters for children within those categories were similar, as assessed by visual inspection. This time, apparently, the infection affected the pulmonary function test parameters more, as two parameters out of four are statistically significantly different between groups. The median values of FEV1 are significantly lower for children with asthma with SARS-CoV-2 infection (0.78) compared to the others (0.88) (U = 3739.50, z = 4.295, and *p* < 0.0005). Median values of FEF_25–75_ are significantly lower for children with SARS-CoV-2 infection (0.70) compared to the others (0.82) (U = 3340.50, z = 2.726, and *p* = 0.006). Also, median values of FeNO are significantly higher for children with asthma with SARS-CoV-2 infection (30.00) compared to those who were not infected (15.00) (U = 1575.00, z = −4.210, and *p* < 0.0005) (Figure 2).

A sign test with continuity correction was conducted to determine the effect of COVID-19 disease on FeNO values and pulmonary function test parameters. These parameters were measured before and after the SARS-CoV-2 infection (Table 11). Of the 79 children who suffered from SARS-CoV-2 infection, the FeNO value increased for 72 participants, decreased for 4 children, and remained the same for 3 children. There was a statistically significant median increase in FeNO values after the children had COVID-19 (30.00) compared to the values before contracting COVID-19 (22.00), z = 8.144 and *p* < 0.0005. For all four pulmonary function test parameters there was mostly a decrease in measured values after SARS-CoV-2 infection, and the identified differences were statistically significant, *p* < 0.05 (Table 11).

For the 67 children who did not contract COVID-19, the evolution of FeNO and pulmonary function test parameters is quite the opposite. The FeNO value decreased for 52 participants, increased for 10 children, and remained the same for 5 children. There was a statistically significant decrease of FeNO median values after the study period (15.00) compared to the values before (23.00), z = −5.207 and *p* < 0.0005. For all four pulmonary function test parameters there was mostly an increase in measured values after the study period, and the reported differences were statistically significant at *p* < 0.05 (Table 11).

Analysis based on a Kruskal–Wallis H test was conducted to determine if there were differences in FeNO and pulmonary function test parameters between children with treatment dose adjustments (parents’ self-management of doses) who had COVID-19 disease or not (Table 12). The results indicated that the median values of FeNO increased for children with a step-down in dose, both for children with COVID-19 and without COVID-19, indicating the influence of the parents’ decision upon the FeNO values. These values were statistically significantly different than the values for children with the same step or with a step-up in dose, both for children with and without SARS-CoV-2 infection. For pulmonary function test parameters, statistically significant differences between values were not identified with *p* > 0.05 (Table 12).

The analysis regarding the distribution of FeNO and pulmonary function test parameters divided by the GINA asthma control levels indicated that children with partially controlled asthma have significantly higher values of FeNO compared to children with well-controlled asthma, both for children who had COVID-19 and for children who did not have COVID-19. For children from the COVID-19 subgroup, there were no other statistically significant differences for the pulmonary parameters between different GINA asthma control levels. For children without COVID-19, the analysis revealed that FEF_25–75_ values were statistically significantly higher for children with partially controlled asthma, *p* = 0.042 (Table 13).

Analysis based on a Mann–Whitney test was conducted to determine if there were differences in FeNO and pulmonary function test parameters between children with different treatment steps modifications by physicians who had COVID-19 disease or not. The results indicated that the median values of FeNO decreased for children with one step-down in dose, both for children with and without COVID-19 disease, but with no statistically significant difference with *p* > 0.05 (Table 14). Similar results were obtained for pulmonary function test parameters, where all values were increased for children with one step-down in dose compared to the other children with asthma, but with no statistically significant differences with *p* > 0.05 (Table 14).

A comparison between non-infected and infected children with asthma is present in Supplementary Materials Table S1.

### 3.4. Baseline and Follow-Up of FeNO and Lung Function Parameters Evolution (Variation Expressed as the Difference Between BEFORE and AFTER Values)—Groups Distribution

A Mann–Whitney U test was run to determine if there were differences in the variation of FeNO and pulmonary function test parameters by gender or residence. Distributions of these parameters for children within the categories were similar, as assessed by visual inspection. Median variation of FeNO and median variation in pulmonary function test parameters were not statistically significantly different between girls and boys, or between children from urban and rural areas with *p* > 0.05 (Table 15).

A similar analysis based on a Kruskal–Wallis test was conducted to determine if there were differences in FeNO and pulmonary function test parameters variations between different age groups. Distributions of FeNO and pulmonary function test parameters variations were similar for all groups, as assessed by visual inspection of a boxplot. No statistically significant differences between the different age groups were identified with *p* > 0.05 (Table 15).

A Mann–Whitney U test was run to determine if there were differences in FeNO and pulmonary function test parameters variations between children who were affected by SARS-CoV-2 infection or not. Distributions of these parameters for children within those categories were similar, as assessed by visual inspection. The median variation in all four parameters and FeNO was statistically significantly different between groups defined by children with and without SARS-CoV-2 infection. Median variation of FeNO was significantly higher for children who contracted COVID-19 disease (6.00 vs. −4.00; U = 315.50, z = −9.162, and *p* < 0.0005). For median pulmonary function test parameters variations, values were significantly lower for children who were infected with SARS-CoV-2 virus: for FVC −0.03 vs. 0.03 (U = 4820.50, z = 8.555, and *p* < 0.0005), for FEV1 −0.05 vs. 0.04 (U = 5143.00, z = 9.828, and *p* < 0.0005), for PEF −0.07 vs. 0.02 (U = 4921.00, z = 8.944, and *p* < 0.0005), and for FEF_25–75_ −0.05 vs. 0.03 (U = 4780.50, z = 8.392, and *p* < 0.0005). In fact, all children with a SARS-CoV-2 infection presented a negative median variation for pulmonary function test parameters, meaning that the measurements after COVID-19 were smaller than the initial measurements, as opposed to children without infection who had positive median variations, meaning higher values. The behavior of FeNO median variation is reversed as children with COVID-19 presented a positive median variation compared to a negative median variation for the other children (Table 15).

Analysis based on a Kruskal–Wallis test was conducted to determine if there were differences in FeNO and pulmonary function test parameters between children with different treatment doses following SARS-CoV-2 infection. Statistically significant differences were observed for median FeNO values as children with a step-down in dose had the highest median variation (8.00), children with the same dose had a negative median variation of −0.50, and children with a step-up in dose had a median variation of −0.00 (χ^2^(2) = 33.087, and *p* < 0.0005). For all four pulmonary function test parameters, median variations were statistically significantly different: children with a step-down in dose had a negative median variation significantly lower than children with the same dose whose values were higher than children with a step-up in dose, with *p* < 0.0005 (Table 15). Children with partially controlled asthma presented a significantly higher positive variation of FeNO compared to children with well-controlled asthma. On the other hand, the same children presented statistically significant lower negative variations in the pulmonary parameters (so values after were lower than the values before), as defined in Table 15.

Similar analysis based on a Mann–Whitney U test reflected the physicians’ adjustments. It was conducted to determine if there were differences in FeNO and pulmonary function test parameters variation between children with different treatment steps following SARS-CoV-2 infection. Statistically significant differences were observed for all parameters. Children with a one step-down in dose had a negative FeNO variation (−3.50), which was statistically significantly lower than children with the same treatment step or a one step-up in dose (2.50), and the borderline *p* value was 0.05. All pulmonary function test parameters had a positive variation (so values after were higher than the values before) for children with one step-down in dose, and a negative variation for the other children, all of which were statistically significantly different with *p* < 0.05 (Table 15).

A Kruskal–Wallis test was conducted to determine if there were differences in FeNO and pulmonary function test parameters variations between children with various numbers of exacerbations per year. Distributions of FeNO and pulmonary function test parameters were similar for all groups, as assessed by visual inspection of a boxplot. Statistically significant differences between the different numbers of exacerbations were identified for all parameters (Table 15).

## 4. Discussion

With the onset of the COVID-19 pandemic, a number of changes have occurred in the medical system and the approach to pediatric emergencies, children with asthma being one of the categories considered at-risk. The safety measures put in place during the lockdown period had the advantage that pediatric patients with asthma were not exposed to daily triggers as often, but they were nevertheless not excluded from SARS-CoV-2 infection.

Among the most effective methods in achieving good asthma control in children, and one which is accepted by the World Health Organization (WHO), is adherence to asthma treatment, but still, only half of them adhered to the treatment prescribed by the physician with inhaled corticosteroids. According to the GINA strategy report guideline the causes of poor adherence to medication are treatment-related, intentional and unintentional factors [18,31,32].

Following SARS-CoV-2 infection, pathological changes occur that alter pulmonary function, which means children with chronic conditions such as asthma or immunodeficiency are at risk. Adherence to asthma treatment during the COVID-19 pandemic has helped obtain a good management of the disease, with studies showing that inhaled corticosteroids have beneficial effects by suppressing inflammatory cytokines and viral replication. Also, it was recommended to avoid the use of nebulizers during the pandemic because they increased the risk of spreading SARS-CoV-2 Conversely, pressurized metered dose inhaler (pMDI) was preferred because of its advantages, namely the use of the device without aid, improvement of the technique through education, and a reduction in the number of hospitalizations [22,33,34,35,36,37,38].

The COVID-19 pandemic led to changes in parents’ perception of the asthma treatment of children as implementing safety measures and a reduction in exposure to risk factors meant that acute manifestations of this chronic lung disease were less frequent. However, adherence to treatment was reduced [36,39,40]. A reduction in the use of ICS during the COVID-19 pandemic was seen in the young population, suggesting that the factors involved in explaining this outcome are related to the difficulty in obtaining a new prescription (unintentional factors) or related to the effects of ICS in lowering immunity (intentional factors), all of which intervene in the decrease in adherence to asthma medication [41].

As can be observed in the present study, some of the parents of pediatric patients with asthma decided to modify the dose of ICS and bronchodilator during the COVID-19 pandemic due to the observed improvement of symptoms, but with long-term consequences on lung function. The median value of FeNO increased in pediatric patients with asthma whose parents initiated a step-down in dose (39.50) on their own initiative compared to those who followed the instructions of the physician (20.50). Regarding pulmonary function parameters analysis, the values tended to decrease in pediatric patients with asthma whose parents’ initiated a step-down in dose and show optimal values in pediatric patients who kept to the recommended dose.

According to the GINA guidance about COVID-19, before reducing the asthma treatment by one step it is necessary that the patient has a good control of the disease, and, in particular, epidemiologic conditions, such as the COVID-19 pandemic, may lead to the decision being made with caution because any change in the dose of the medication may increase the risk of infection with SARS-CoV-2 and an increase in the number of asthma exacerbations. The parents’ perception of the disease is also a reason why adherence to asthma treatment is not exactly satisfactory because they notice that their child’s symptoms are improved, which is misleading, and thus intervene in the treatment without a routine check-up to assess lung function [22,42,43,44,45]. Moreover, studies show that a continuous collaboration between the physician and the patient would help to achieve an adequate adherence to asthma treatment with beneficial effects such as reducing the number of exacerbations, avoiding the process of airway remodeling, and keeping symptoms under control [46,47,48,49,50,51].

According to the results of the present study, the number of exacerbations was higher in children who had a step-down in dose modified by their parents compared to children with asthma whose parents decided to follow the indications of the physician or who step-up in dose, where the number of exacerbations was lower.

The level of asthma control is quite important because studies have been reported showing that pediatric patients with controlled asthma were less likely to develop COVID-19 than uncontrolled patients with asthma where the rate of SARS-CoV-2 infection and hospitalizations were higher [52,53,54,55]. Chronic airway inflammation is one of the pathophysiologic features of asthma that may persist despite good airway control. It has been shown to be useful to assess pulmonary function parameters and not only symptoms at the time of the initiation of asthma treatment, so that any change in ICS dose may lead to a decrease in pulmonary function test parameters [56,57,58].

Changes in pulmonary function were also observed after SARS-CoV-2 infection, especially in children who showed persistent symptoms even after a longer period of time after infection, with lower FEV1 and FVC values compared to patients who did not have COVID-19 disease. Patients were also evaluated for nitric oxide in exhaled air, with increased values found after SARS-CoV-2 infection [59,60,61].

In the present study, changes in pulmonary function were observed in both COVID-19 infected and non-infected pediatric patients with asthma. For pediatric patients with asthma with COVID-19, median FeNO values were elevated and pulmonary function parameters were reduced after SARS-CoV-2 infection. For pediatric patients who did not have COVID-19, median FeNO values decreased while pulmonary function parameters increased, indicating a favorable evolution of lung function over time. This aspect may be influenced by the safety measures implemented during lockdown period, adherence to treatment recommended by the physician, and exposure to specific allergens.

One of the most common triggers is acute respiratory tract infections, which can also cause asthma exacerbations. Also, peak expiratory flow (PEF) is a useful biomarker in the assessment of pulmonary function in patients with asthma [62,63]. It was found that a decrease in PEF by 20% or more of the predicted value will influence the level of control of asthma and the occurrence of asthma exacerbations. Also, increasing the dose of ICS at the first signs of an exacerbation may be beneficial in relieving acute symptoms and increasing PEF [64,65].

Children with asthma whose parents opted to step-down in dose had lower PEF values (0.655) compared to children with asthma whose parents opted to step-up in dose (0.710) or remain at the same treatment regimen prescribed by the physician (0.715), which had higher PEF values. The present study showed modified PEF values in children with asthma who were infected with the SARS-CoV-2 virus, and the median PEF values lowered after contracting COVID-19.

For better symptom control so that patients with asthma are not at risk of triggering more episodes of asthma exacerbations, studies have shown that a combination of an inhaled corticosteroid and a long-acting bronchodilator is preferable to the administration of a bronchodilator as reliever alone (SABA) [66,67,68,69].

Asthma is a complex pathology that requires careful monitoring and continuous adaptation of treatment regimens in order to obtain better disease control. The combination of ICS–formoterol is recommended for use in pediatric patients with asthma in Steps 3–5. This strategy, called MART (maintaining and reliever therapy), has shown much more satisfactory effects in terms of symptom control, as well as better adherence to treatment due to the fact that it is no longer necessary to introduce a SABA into the treatment plan as needed [18,70].

It can be seen that a lower number of exacerbations is associated with a constant collaboration between parent and physician because children with asthma whose parents kept the same dose of treatment as recommended had better control of the disease. However, children with asthma who showed partial control of the disease were those whose parents reduced the treatment step recommended by the physician, and for them exacerbation has been occurring more frequently. These aspects show the impact that the COVID-19 pandemic has had on asthma management in children, emphasizing the importance of maintaining effective parent–clinician collaboration in the context of a global health emergency.

### Limitations of the Study

Due to the fact that this study is retrospective, the absence of behavioral or psychosocial data, socioeconomic status, or parental education data that would justify the choices made by parents during the pandemic and post-pandemic period in the case of asthma treatment of children must be taken into account.

It is necessary to specify the relatively small sample size of the study because not all pediatric patients with asthma were medically monitored during the COVID-19 pandemic due to the restrictions of that period, thus interfering with data collection. Another limitation of the study is the relatively small number of patients diagnosed with asthma who tested positive for COVID-19, and presented for routine follow-up for evaluation of lung function, and had changes in asthma medication both before and after SARS-CoV-2 infection.

This study provides valuable information on how the COVID-19 pandemic and SARS-CoV-2 virus infection influenced the decisions of parents of children with asthma on their treatment, and how pulmonary function was affected. Further research involving a broader study population of children with asthma is necessary, ensuring more accurate assessment for improving asthma control through optimal treatment which is aligned with medical principles shaped by specific epidemiological conditions.

## 5. Conclusions

Following this research, an increase in FeNO values and modifications in pulmonary function test parameters values were observed in children with asthma who were infected with SARS-CoV-2, and the changes were influenced by parental dose adjustments. The pandemic significantly altered asthma management practices among caregivers, with measurable effects on clinical outcomes. Although beneficial effects have been observed in children with asthma during the COVID-19 pandemic due to limited contact with numerous triggers, the effects that SARS-CoV-2 infection had on the pulmonary function of these patients should not be minimized.

This study highlights the effects that the COVID-19 pandemic had on asthma treatment in the pediatric population and what consequences were observed in lung function. A permanent collaboration between the parent/caregiver/pediatric patient and the attending physician, a constant evaluation of the symptoms, compliance with the asthma treatment with ICS and a bronchodilator, ensuring the comfort of the child, as well as the need for enhanced parent/caregiver education and close clinical monitoring during public health emergencies, are factors that would help to achieve good control of the disease.

## Figures and Tables

**Table 1:** Baseline distribution of FeNO and lung function parameters by gender, residence, and age group.

	Parameter		Gender			Residence			Age groups (Years Old)			
			F	M	*p* *	Urban	Rural	*p* *	<6	6–11	12–17	*p* **
BEFORE	FeNO	Median values	22.00	23.00	0.875	23.00	22.00	0.378	21.00	23.00	31.00	0.001 ^#^
	FVC		0.94	0.90	0.209	0.900	0.930	0.208	0.900	0.920	0.970	0.999
	FEV1		0.845	0.835	0.554	0.835	0.860	0.134	0.830	0.840	0.900	0.318
	PEF		0.745	0.730	0.797	0.720	0.755	0.133	0.735	0.690	0.890	0.018 ^#^
	FEF_25–75_		0.785	0.790	0.449	0.775	0.805	0.267	0.790	0.780	0.870	0.065

* Mann–Whitney U test. ** Kruskal–Wallis H test. ^#^ Statistically significant.

**Table 2:** Baseline distribution of FeNO and lung function parameters by treatment step.

	Parameter		Step				
			Step 1	Step 2	Step 3	Steps 4–5	*p* *
BEFORE	FeNO	Median values	22.00	24.00	21.00	20.50	0.793
	FVC		0.930	0.910	0.900	0.970	0.753
	FEV1		0.850	0.840	0.810	0.890	0.675
	PEF		0.760	0.720	0.730	0.710	0.715
	FEF_25–75_		0.840	0.780	0.795	0.870	0.398

* Kruskal–Wallis H test.

**Table 3:** Follow-up distribution of FeNO and lung function parameters by gender, residence, and age group for Study Group 1 and Study Group 2.

	Parameter		Gender			Residence			Age Groups (Years Old)			
			F	M	*p* *	Urban	Rural	*p* *	< 6	6–11	12–17	*p* **
AFTER	FeNO	Median values	26.50	25.00	0.581	25.00	25.50	0.497	4.00	23.50	31.00	0.010 ^#^
	FVC		0.940	0.915	0.370	0.910	0.945	0.449	0.9	0.93	0.93	0.947
	FEV1		0.830	0.845	0.770	0.820	0.865	0.316	0.825	0.82	0.875	0.386
	PEF		0.700	0.700	0.776	0.685	0.715	0.288	1.025	0.68	0.73	0.014 ^#^
	FEF_25–75_		0.755	0.765	0.781	0.750	0.775	0.521	0.78	0.74	0.83	0.033

* Mann–Whitney U test. ** Kruskal–Wallis H test. ^#^ Statistically significant.

**Table 4:** Follow-up distribution of FeNO and lung function parameters by dose adjustments, self-managed by parents without consulting the physicians for Study Group 1 and Study Group 2.

	Parameter		Self-Management of Medication Doses Evolution (by Parents)				Self-Management of Medication Adjustments (by Parents)		
			Step-Down	Same	Step-Up	*p* *	Yes	No	*p* **
AFTER	FeNO	Median values	39.50	20.50	19.50	<0.0005 ^#^	29.50	19.50	0.001 ^#^
	FVC		0.880	0.945	0.935	0.121	0.925	0.935	0.633
	FEV1		0.780	0.870	0.850	0.025 ^#^	0.825	0.850	0.399
	PEF		0.655	0.715	0.710	0.157	0.695	0.710	0.714
	FEF_25–75_		0.705	0.805	0.770	0.123	0.755	0.770	0.463

* Kruskal–Wallis H test. ** Mann–Whitney U test. ^#^ Statistically significant.

**Table 5:** Treatment step evolution by physician correlated with parents’ self-management of doses for Study Group 1 and Study Group 2.

Study Variable	Category	Treatment Step Evolution (by Physician)		Total	*p* *
		One Step-Down	Same Step or One Step-Up		
		10 (6.85%)	136 (93.15%)	146 (100%)	-
Parents self-management of doses	Step-down	0 (0%)	42 (100%)	42 (100%)	0.019
		0%	30.9%		
	Same	4 (6.1%)	62 (93.9%)	66 (100%)	
		40%	45.6%		
	Step-up	6 (15.8%)	32 (84.2%)	38 (100%)	
		60%	23.5%		

* Chi Square test. Values presented in light gray represent the sum of values by column.

**Table 6:** GINA asthma control levels.

Study Variable	Category	GINA Asthma Control Levels		Total	*p* *
		Well-Controlled	Partially Controlled		
		72 (49.30%)	74 (50.70%)	146 (100%)	-
Parents self-management of doses	Step-down	8 (19.05%)	34 (80.95%)	42 (100%)	<0.0005 *
		11.11%	45.95%		
	Same	45 (68.18%)	21 (31.82%)	66 (100%)	
		62.50%	28.37%		
	Step-up	19 (50%)	19 (50%)	38 (100%)	
		26.39%	25.68%		

* Chi-Square test. Values presented in light gray represent the sum of values by column.

**Table 7:** Follow-up distribution of FeNO and lung function parameters following GINA asthma control levels.

	Parameter		GINA Asthma Control Levels		
			Well-Controlled	Partially Controlled	*p* *
AFTER	FeNO	Median values	13.50	36.00	<0.0005 ^#^
	FVC		0.930	0.930	0.507
	FEV1		0.850	0.825	0.185
	PEF		0.720	0.690	0.201
	FEF_25–75_		0.770	0.750	0.627

* Mann–Whitney U test. ^#^ Statistically significant.

**Table 8:** Follow-up distribution of FeNO and lung function parameters by treatment step evolution by physicians.

	Parameter		Treatment Step Evolution		
			One Step-Down	Same Step or One Step-Up	*p* *
AFTER	FeNO	Median values	9.00	26.00	0.008 ^#^
	FVC		0.990	0.925	0.093
	FEV1		0.915	0.820	0.004 ^#^
	PEF		0.780	0.690	0.055
	FEF_25–75_		0.880	0.750	0.075

* Mann–Whitney U test. ^#^ Statistically significant.

**Table 9:** Follow-up distribution of FeNO and lung function parameters by exacerbations.

	Parameter		Exacerbations				
			None	1/Year	2/Year	3/Year	*p* *
AFTER	FeNO	Median values	11.00	29.50	37.00	37.00	<0.0005 ^#^
	FVC		0.940	0.930	0.930	0.870	0.815
	FEV1		0.850	0.830	0.840	0.730	0.286
	PEF		0.700	0.670	0.730	0.640	0.429
	FEF_25–75_		0.770	0.710	0.790	0.665	0.190

* Kruskal–Wallis H test. ^#^ Statistically significant.

**Table 10:** Distribution of exacerbations by doses adjustments, self-managed by parents without consulting the physicians and treatment step evolution.

Study Variable	Category	Exacerbations				Total	*p* *
		None	1/Year	2/Year	3/Year		
Parents self-management of doses	Step-down	1 (2.38%)	20 (47.62%)	16 (38.1%)	5 (11.9%)	42 (100%)	
		1.96%	35.72%	51.61%	62.5%		<0.0005 ^#^
	Same	33 (50%)	18 (27.27%)	12 (18.18%)	3 (4.55%)	66 (100%)	
		64.71%	32.14%	38.71%	37.5%		
	Step-up	17 (44.74%)	18 (47.37%)	3 (7.89%)	0 (0%)	38 (100%)	
		33.33%	32.14%	9.68%	0%		
Treatment step evolution by physician	One step-down	6 (60%)	4 (40%)	0 (0%)	0 (0%)	10 (100%)	0.187
		11.76%	7.14%	0%	0%		
	Same step or one step-up	45 (33.09%)	52 (38.24%)	31 (22.79%)	8 (5.88%)	136 (100%)	
		88.24%	92.86%	100%	100%		

* Chi-Square test. Values presented in light gray represent the sum of values by column. ^#^ Statistically significant.

**Table 11:** Evolution of FeNO and lung function parameters in children with asthma who contract and did not contract COVID-19.

	Parameter	Median		Evolution (BEFORE Values–AFTER Values) Number of Children				
		BEFORE	AFTER	Increased	Decrease	Tie	z	*p* *
COVID-19	FeNO	22.00	30.00	72	4	3	8.144	<0.0005 ^#^
	FVC	0.94	0.90	8	61	10	−6.260	<0.0005 ^#^
	FEV1	0.82	0.78	4	74	1	−7.813	<0.0005 ^#^
	PEF	0.75	0.70	1	76	2	−8.433	<0.0005 ^#^
	FEF_25–75_	0.78	0.70	2	73	4	−8.083	<0.0005 ^#^
Non-COVID-19	FeNO	23.00	15.00	10	52	5	−5.207	<0.0005 ^#^
	FVC	0.90	0.94	58	7	2	6.202	<0.0005 ^#^
	FEV1	0.85	0.88	56	8	3	5.875	<0.0005 ^#^
	PEF	0.69	0.70	46	19	2	3.225	0.001 ^#^
	FEF_25–75_	0.80	0.82	51	14	2	4.465	<0.0005 ^#^

* Sign test. ^#^ Statistically significant.

**Table 12:** Distribution of FeNO and lung function parameters of children with asthma after their parents’ self-managed the treatment.

	Parameter		Parents Self-Management of Doses			*p* *
			Step-Down	Same	Step-Up	
COVID-19	FeNO	Median values	38.00	29.50	25.00	0.001 ^#^
	FVC		0.890	0.875	0.990	0.059
	FEV1		0.780	0.730	0.840	0.077
	PEF		0.690	0.630	0.750	0.069
	FEF_25–75_		0.690	0.690	0.800	0.130
Non-COVID-19	FeNO	Median values	46.50	13.00	13.00	0.005 ^#^
	FVC		0.880	0.980	0.940	0.140
	FEV1		0.835	0.890	0.880	0.382
	PEF		0.595	0.720	0.700	0.094
	FEF_25–75_		0.785	0.870	0.810	0.797

* Kruskal–Wallis H test. ^#^ Statistically significant.

**Table 13:** Distribution of FeNO and pulmonary function test parameters following assessment of asthma control at clinical visits for COVID-19 and non-COVID-19 pediatric patients.

	Parameter		GINA Asthma Control Levels		
			Well-Controlled	Partially Controlled	*p* *
COVID-19	FeNO	Median values	23.50	36.00	0.001 ^#^
	FVC		0.900	0.900	0.886
	FEV1		0.800	0.780	0.810
	PEF		0.725	0.680	0.124
	FEF_25–75_		0.755	0.700	0.224
Non-COVID-19	FeNO	Median values	9.50	36.00	<0.0005 ^#^
	FVC		0.945	0.940	0.926
	FEV1		0.895	0.880	0.687
	PEF		0.700	0.700	0.932
	FEF_25–75_		0.790	0.870	0.042 ^#^

* Mann–Whitney U test. ^#^ Statistically significant.

**Table 14:** Distribution of FeNO and lung function parameters by treatment step modifications by physicians and COVID-19 distribution.

	Parameter		Treatment Step Evolution		
			One Step-Down	Same Step or One Step-Up	*p* *
COVID-19	FeNO	Median values	8.00	30.50	0.203
	FVC		0.960	0.900	0.759
	FEV1		0.850	0.780	0.658
	PEF		0.800	0.695	0.405
	FEF_25–75_		0.760	0.700	0.785
Non-COVID-19	FeNO	Median values	9.00	15.00	0.224
	FVC		1.000	0.940	0.198
	FEV1		0.920	0.870	0.067
	PEF		0.760	0.680	0.098
	FEF_25–75_		0.890	0.805	0.225

* Mann–Whitney U test.

**Table 15:** Variation of FeNO and lung function parameters (values computed as before–after).

Parameter	Category	FeNO (Median Variation)	Pulmonary Function Test Parameters (Median Variation)			
			FVC	FEV1	PEF	FEF_25–75_
Gender	F	2.00	0.00	−0.03	−0.05	−0.03
	M	2.00	0.00	−0.02	−0.03	−0.01
	*p*	0.655 *	0.795 *	0.473 *	0.413 *	0.51 *
Residence	Urban	2.00	0.00	−0.02	−0.03	−0.01
	Rural	2.00	0.00	−0.02	−0.05	−0.03
	*p*	0.764 *	0.551 *	0.897 *	0.355 *	0.578 *
Age groups	< 6	0.00	−0.03	−0.01	0.12	−0.03
	6–11	2.00	0.00	−0.02	−0.04	−0.02
	12–17	1.00	0.01	−0.01	−0.03	−0.01
	*p*	0.595 **	0.633 **	0.872 **	0.457 **	0.174 **
Parent self-management of doses	Step-down	8.00	−0.03	−0.04	−0.06	−0.05
	Same step	−0.50	0.02	0.02	−0.01	0.00
	Step-up	0.00	0.01	0.01	−0.02	−0.01
	*p*	<0.0005 **^,#^	<0.0005 **^,#^	<0.0005 **^,#^	<0.0005 **^,#^	0.001 **^,#^
Treatment step evolution	One step down	−3.50	0.04	0.04	0.02	0.03
	Same step or one step up	2.50	−0.01	−0.03	−0.04	−0.02
	*p*	0.05 *^,#^	0.003 *^,#^	0.005 *^,#^	0.008 *^,#^	0.021 *^,#^
Exacerbations	None	−4.00	0.02	0.02	0.01	0.01
	1/year	4.50	−0.02	−0.04	−0.05	−0.04
	2/year	4.00	0.00	−0.03	−0.04	−0.01
	3/year	6.00	−0.04	−0.05	−0.04	−0.05
	*p*	<0.0005 **^,#^	<0.0005 **^,#^	<0.0005 **^,#^	<0.0005 **^,#^	<0.0005 **^,#^
COVID-19 disease	Yes	6.00	−0.03	−0.05	−0.07	−0.05
	No	−4.00	0.03	0.04	0.02	0.03
	*p*	<0.0005 *^,#^	<0.0005 *^,#^	<0.0005 *^,#^	<0.0005 *^,#^	<0.0005 *^,#^
GINA asthma control levels	Well-controlled	−1.00	0.01	0.01	−0.01	0
	Partially controlled	5.00	−0.01	−0.03	−0.04	−0.03
	*p*	<0.0005 *^,#^	0.002 *^,#^	0.010 *^,#^	0.001 *^,#^	0.027 *^,#^

* Mann–Whitney U test. ** Kruskal–Wallis H test. ^#^ Statistically significant.

## Data Availability

The authors declare that the data of this research are available from the corresponding authors upon reasonable request.

## Supplementary Materials

The following supporting information can be downloaded at: https://www.mdpi.com/article/10.3390/jcm14103289/s1. Table S1: Distribution of FeNO and lung function parameters by the number of exacerbations and COVID-19 distribution.
