# Supplementary material for: Impact of the COVID-19 Pandemic on Lung Function and Treatment Decisions in Children with Asthma: A Retrospective Study
Source: J Clin Med. 2025 May 8;14(10):3289. doi: 10.3390/jcm14103289 (PMC12111926; doi:10.3390/jcm14103289)
Supplement: Supplementary file 1 [file jcm-14-03289-s001.zip › jcm-3582454-supplementary.pdf]

Impact of the COVID-19 Pandemic on Lung Function and Treatment Decisions in Children with Asthma: A Retrospective Study

Comparison between non-infected and infected children with asthma

An analysis based on a Kruskal-Wallis H test was conducted to determine if there were differences in FeNO and pulmonary function tests parameters between children who suffered from exacerbations and who had COVID-19 disease or not (Table S1). For the children who were not infected with SARS-CoV-2 virus, the median values of FeNO were statistically significantly different between the different number of exacerbations per year, increasing from the value 10.00 (for children with no exacerbation), up to 75.00 (for children with 3 exacerbations / year), p < 0.0005. No other statistically significant differences were identified.

Table S1. Distribution of FeNO and lung function parameters by the number of exacerbations and COVID-19 distribution.

	Parameter		Exacerbations				
			None	1 / year	2 / year	3 / year	p *
COVID-19	FeNO	Median values	23.00	30.00	36.00	37.00	0.119
	FVC		0.885	0.925	0.880	0.855	0.387
	FEV1		0.800	0.795	0.780	0.710	0.287
	PEF		0.725	0.655	0.730	0.595	0.682
	FEF_{25-75}		0.760	0.700	0.740	0.605	0.339
Non-COVID-19	FeNO	Median values	10.00	24.50	46.50	75.00	< 0.0005[#]
	FVC		0.95	0.955	0.935	1.095	0.419
	FEV1		0.85	0.885	0.9	0.95	0.636
	PEF		0.67	0.695	0.725	0.72	0.630
	FEF_{25-75}		0.8	0.865	0.86	0.815	0.387

* Kruskal-Wallis H test. [#] Statistically significant.
